# Molecular Mechanisms of Taste Recognition: Considerations about the Role of Saliva

**DOI:** 10.3390/ijms16035945

**Published:** 2015-03-13

**Authors:** Tibor Károly Fábián, Anita Beck, Pál Fejérdy, Péter Hermann, Gábor Fábián

**Affiliations:** 1 Private Practitioner, Ludvigsmindevej 14, st. Faaborg DK-5600, Denmark; 2 Clinic of Pediatric Dentistry and Orthodontics, Faculty of Dentistry, Semmelweis University Budapest, Szentkirályi utca 47, Budapest H-1088, Hungary; E-Mails: beck.anita@dent.semmelweis-univ.hu (A.B.); fabian.gabor@dent.semmelweis-univ.hu (G.F.); 3 Clinic of Prosthetic Dentistry, Faculty of Dentistry, Semmelweis University Budapest, Szentkirályi utca 47, Budapest H-1088, Hungary; E-Mails: fejerdy.pal@dent.semmelweis-univ.hu (P.F.); hermann.peter@dent.semmelweis-univ.hu (P.H.)

**Keywords:** taste, perception, receptor, saliva, salivary

## Abstract

The gustatory system plays a critical role in determining food preferences and food intake, in addition to nutritive, energy and electrolyte balance. Fine tuning of the gustatory system is also crucial in this respect. The exact mechanisms that fine tune taste sensitivity are as of yet poorly defined, but it is clear that various effects of saliva on taste recognition are also involved. Specifically those metabolic polypeptides present in the saliva that were classically considered to be gut and appetite hormones (*i.e.*, leptin, ghrelin, insulin, neuropeptide Y, peptide YY) were considered to play a pivotal role. Besides these, data clearly indicate the major role of several other salivary proteins, such as salivary carbonic anhydrase (gustin), proline-rich proteins, cystatins, alpha-amylases, histatins, salivary albumin and mucins. Other proteins like glucagon-like peptide-1, salivary immunoglobulin-A, zinc-α-2-glycoprotein, salivary lactoperoxidase, salivary prolactin-inducible protein and salivary molecular chaperone HSP70/HSPAs were also expected to play an important role. Furthermore, factors including salivary flow rate, buffer capacity and ionic composition of saliva should also be considered. In this paper, the current state of research related to the above and the overall emerging field of taste-related salivary research alongside basic principles of taste perception is reviewed.

## 1. Introduction

The gustatory system plays a critical role in determining food preferences and food intake [1] and, consequently, one’s the nutritive, energy and electrolyte balance. The sense of taste is devoted primarily to evaluating the quality of nutrients and distinguishing between safe and dangerous foods [2,3]. On the basis of taste perception, further food intake is then considered to be, or not to be, desirable [1]. Although aided by smell and visual inspection, the final recognition and selection relies primarily on the chemoreceptive events occurring in the mouth [2]. Taste cells detect sugars, amino acids, poisons, acids, and minerals which tastants are usually cues for sweet, umami, bitter, sour, and salty tastes, respectively [4]. These five taste qualities are called basic tastes because each of them has distinct individual taste and are believed to be detected by different taste cells [4]. Sweet, umami [3] and salty [1] are typically associated with palatability, thus inducing acceptance behavior and initiating digestive physiological responses [3]. Sweet, umami and salt modalities allow recognition of energy-containing nutrients and maintenance of electrolyte balance [1]. By contrast, bitter taste likely acts as a warning mechanism against toxic or harmful chemicals [3,5], even if humans regularly choose to ingest natural and synthetic bitter-tasting compounds in foods beverages and medications [5]. To prevent ingestion of toxins, priority is placed on detecting, rather than discriminating, bitter-tasting compounds [5], thereby inducing evoking signals and consequent avoidance, rejection or spitting out of potential food toxins before swallowing [3,5]. Besides bitter taste, sour taste modality is thought to act as brake or warning against noxious foods [1].

There are also various complementary taste modalities like “fat-taste” or “fatty acid taste” [6,7], “CO_2_ taste” [8] and the taste-related sensation “astringency” [9,10]. Related studies in humans strongly indicate that fat/fatty acid-sensing mechanisms may contribute to overeating and obesity [6,11,12,13,14,15]; the sensation of astringency has been proposed as representing a warning cue that discourages the ingestion of foods containing overly high concentrations of (poly)phenolic compounds [10,16]; whereas CO_2_ taste is understood to be a mechanism for recognizing CO_2_-producing sources so as to, for instance, avoid fermenting foods [8].

## 2. Taste Buds

Taste buds are clusters of 50–80 (up to 100) polarized (bipolar) neuroepithelial cells that form compact, columnar pseudostratified “islands” embedded in the surrounding oral epithelium [17,18]. There are roughly between 2000 and 5000 taste buds in the human oral cavity (and surrounding the oral region), distributed on the tongue, the palate, and to a lesser extent the epiglottis, pharynx, and larynx [19,20,21,22]. Taste buds located on the anterior tongue are embedded in the fungiform papillae [22]. These taste buds are innervated by the chorda tympani nerve, a branch of the facial nerve [22]. Roughly 30% of the tongue’s taste buds are located here [21]. Taste buds in the posterior tongue are located in the circumvallate papillae [22]. These taste buds are innervated by the glossopharyngeal nerve [22]. Roughly 40% of the tongue’s taste buds are located here [21]. There are also taste buds buried in folds in the lateral sides of the tongue, in the foliate papillae [22]. These taste buds are innervated by branches of the chorda tympani and the glossopharyngeal nerve [22]. Roughly 30% of the tongue’s taste buds are located here [21]. Taste buds in the palate are innervated by the greater superficial petrosal nerve, which is another branch of the facial nerve [22], whereas taste buds located in the epiglottis, pharynx, and larynx are innervated by the vagus nerve.

The taste-sensing signals from taste buds (from taste cell receptors) are transferred through peripheral processes of unipolar nerve cells located in the genicular ganglion of the facial nerve (enter the tongue via chorda tympani and n. lingualis), the inferior ganglion of the glossopharyngeal nerve and the inferior ganglion of the vagus nerve [23]. Through these cells, the impulses reach the central nervous parts of the gustatory apparatus, which include the tractus solitarius, the nucleus of the tractus solitarius and its ascendent connections, including the nucleus ventralis posterior medialis of the thalamus, the antero-inferior part of the sensorimotor cortex and the insula [23], as well as several other connections to numerous hypothalamic nuclei and the limbic system [23]. Relayed fibers certainly also reach the frontal cortex.

Although taste buds in all regions respond to sweet, salty, sour, bitter and very likely also to umami (though this has not been studied as thoroughly), there are differences in their sensitivities to these tastes [22,24]. Presumably, these differences reflect different distributions of various taste cell populations from region to region [22] and also differences in sensitivities of taste bud cells present in the various regions [25]. Historically, taste bud cells have been assorted into three major groups on the basis of their morphological phenotypes: Type I, II, and III cells which are also referred to as “dark,” “light,” and “intermediate” cells, respectively [26]. These morphological differences also correlate with functional differences between each of these cell types [4]. Although, many authors referred to all these cells as “taste *receptor* cells” earlier, it is now abundantly clear that roughly half or fewer of all these cells are indeed receptors only [18]. Besides type I, II, and III cells, there are also basal cells in the taste buds. These basal cells are nonpolarized, presumably undifferentiated cells sometimes also termed type IV cells. In contrast to type I, II and III cells, basal cells do not extend processes into the taste pore and are likely to be undifferentiated or immature taste cells [26]. Their significance as a cell population remains to be elucidated [17].

Despite type I cells being the most abundant cells of taste buds, their functions are not well characterized [4,17,18]. It may be considered that the function of these cells is to maintain the structure of taste buds [1]. Type I cells also appear to be involved in terminating synaptic transmission and restricting the spread of transmitters (a role performed in the central nervous system by glial cells); thus, type I cells may also considered to function as “glia in the taste buds” [17,18]. Their role in salty taste sensation was also speculated recently [17,27,28]; however, it remains unclear whether or not these cells have a function in detecting tastants and/or modulating taste stimuli [4,17,18]. Type II taste cells express G protein-coupled receptors (GPCRs) responsible for the detection of sweet, umami, and bitter compounds [1,17,29]; consequently, type II taste cells have at least three subsets of cells that respond to each different stimuli [4]. Interestingly, type II taste cells do not form ultrastructurally identifiable synapses with those nerve fibers (presumably gustatory afferents), which are closely apposed to these cells; consequently, the signals transmitted from these cells to the sensory afferents must be based on unique (and not yet fully characterized) mechanisms [17]. In contrast to type II taste cells, there is consensus that type III cells express proteins associated with synapses and that they form synaptic junctions with (conceivably gustatory afferent) nerve terminals [17]. Type III taste cells are thought to express sour taste receptors and detect sour taste [30]. Although type III cells are presumably the cells responsible for signaling sour taste sensations [17], another key feature of these cells is that they receive input from and integrate signals generated by the type II cells [17,31]. Thus, these cells are not tuned to specific taste qualities but instead respond to a broad spectrum of taste stimuli [17,31]. It is likely that type II cells are also involved in the sensation of salty taste in such an aspecific way [17,31]. However, even though the role of type I taste cells in the detection of salty taste has recently been speculated [17,27,28], it is not yet known which cells in the taste bud are specifically responsible for the salty taste sensation [4,17,22].

Interestingly, cells expressing sweet/umami (T1R) and/or bitter (T2R) and/or sour (*i.e.*, ASICs) taste receptors (and their signaling molecules) have also been reported in several extraoral sites such as the gastrointestinal system [4,32] (including the stomach [33], the gut [34,35], the pancreas [36], and the liver [36]); the respiratory system [37]; the urogenital system [38]; the reproductive system [39,40]; and the brain [41]. Recently, expression of functional umami taste receptor (T1R1/T1R3) was also found in neutrophils [42]. These data support a model where chemosensing mechanisms are conserved throughout the alimentary canal [43]; and also strongly support the concept that cells expressing taste receptors may exert certain “non-gustatory” functions, which might vary according to the extraoral sites of these cells [3,4,5,40,44,45].

## 3. Taste Receptors

In general, sweet, salty, sour, bitter, and umami are considered to be the basic taste qualities. Recent studies have proposed candidate receptors for these five basic tastes [46,47,48] which are divided into two groups: G-protein-coupled receptors (GPCRs) and channel-type receptors [17,48]. The expression patterns of these receptors in taste bud cells suggest that each taste quality may be encoded by a separate population of cells, and these separate populations of taste cells respond to one of the five basic taste stimuli only [49]. Activation of taste cells lead to transmitter release and consequent activation of gustatory nerve fibers [17,18,48]. Response characteristics of the various taste bud cells and their related gustatory nerve fibers are very similar; however, it is very likely that gustatory nerve fibers selectively innervate their corresponding types of taste cells [50]. Thus, taste qualities may be discriminated primarily at the taste cell level [48].

Interestingly, only a few taste bud cells have synaptic contact with nerve fibers in the taste buds [51]. It is likely that sour taste receptor cells (*i.e.*, cells expressing PKD2L1) possess synaptic structures [52] and may use serotonin for synaptic transmission [17,18,48,52,53]. In contrast, taste bud cells expressing receptors and transduction components for sweet, bitter and umami taste do not possess conventional synaptic structures, although they also have close contact with sensory nerve fibers [17,18]. Regarding the signal transmission from these taste cells to gustatory nerve fibers adenosine 5'-trisphosphate (ATP) is the most likely candidate transmitter [17,18], because these taste cells release ATP in response to taste stimuli, respectively [54,55]. The mechanism for the signal transmission from salt-sensitive taste cells to gustatory nerve fibers is not yet known [48].

Although the majority (60%–70%) of taste cells respond exclusively to one of the basic taste qualities [56,57], there is still a significant portion of taste cells that respond to multiple taste qualities [17,31,48]. These cells may contribute to the discrimination of more slight differences between taste compounds [48].

Sweet and umami taste are recognized by the T1R receptor family (T1R1, T1R2 and T1R3) that belongs to family C of GPCRs [3,48]. T1Rs assemble into heterodimeric receptor complexes to function as sweet (T1R2_T1R3) or umami (T1R1_T1R3) taste receptors [48,58,59]; thus the T1R2/T1R3 heterodimer is activated by various sweeteners (*i.e.*, sugars, artificial sweeteners, sweet amino acids, and sweet proteins), whereas the T1R1/T1R3 heterodimer is activated primarily by monosodium l-glutamate in humans [60] and by amino acids in animals (*i.e.*, mouse) [48,59,61]. Although monosodium l-glutamate (MSG) can be considered as the prototypic umami stimulus in humans [60,62,63], umami taste can also be elicited by a few other amino acids (*i.e.*, aspartate), many short peptides, some organic acids (*i.e.*, lactic, succinic, and propionic acids), and expectably also by other compounds [62]. Importantly, umami taste can be enhanced by monophosphate esters of guanosine or inosine nucleosides such as inosine 5'-monophosphate (IMP) and guanosine 5' monophosphate (GMP) [62,63].

T1R receptor-independent sweet and/or umami taste receptors may also exist in taste cells [48]. A potential candidate for umami taste receptors other than T1R1/T1R3 are, for example, mGluR variants such as taste mGluR1 and 4 [25,64,65], which have been shown to be expressed in taste cells [64,65]. These candidate molecules are variants of brain-expressed metabotropic glutamate receptors 1 and 4. These variants (also referred to as “truncated mGluR1” and “taste-mGluR4” [25]) have truncated *N*-terminals to which glutamate still binds, albeit with reduced affinity compared to those receptors expressed in the brain [25,63]. Conceivably, the truncation adapted the receptor to the higher glutamate concentration in the mouth (*i.e.*, in the food) compared to the circumstances in the brain [63,64].

Recent studies have shown that sensitivity of sweet taste cells can be modulated by hormones and other endogenous factors [66] also directly via receptors of taste cells. The endogen mediator leptin (which reduces food intake by acting on hypothalamic receptors) selectively suppresses sweet taste via leptin receptors (Ob-Rb) of taste cells [48]. Similarly, endocannabinoids (that stimulate food intake via cannabinoid receptors mainly in the hypothalamus) enhance sweet taste sensitivity of taste cells via CB1 receptors [48].

Bitter taste is recognized by the T2R receptor family that belongs to family A of GPCRs [48]. In humans, 25 members of the T2R family may function as bitter taste receptors [3,5,48,67]. Certain T2Rs are broadly tuned, being activated by a variety of bitter substances that can be structurally divergent, whereas others are more discretely or narrowly tuned by recognizing a few or single compounds only [3,5]. Although bitter taste is evoked by perhaps tens of thousands of synthetic and natural compounds [5], bitter ligands for some of the T2Rs (*i.e.*, T2R41, T2R42, T2R45, T2R48, T2R60) are still unknown [48,67]. T2Rs are coexpressed in a subpopulation of taste receptor cells [68,69], possibly indicating that T2Rs may also form heterooligomers similarly to T1Rs. However, the functional significance of oligomerization has not yet been elucidated [48,70].

Sweet, umami, and bitter tastes are recognized by different GPCRs but use a common signaling pathway after activation of these receptors [48,71,72]: tastants binding to sweet, umami, and bitter receptors activate the heterotrimeric G-protein (subunits alpha-gustducin, transducin) → phospholipase C β2 (PLCβ2) → inositol-1,4,5-triophosphate (IP3) → Ca^2+^ signaling pathway [3,21,25,48,71,72]. Then the released Ca^2+^ stimulates transient receptor potential channel M5 (TRPM5) which depolarizes the taste cell leading to the generation of action potentials by means of the voltage-gated sodium channels (VGSC) of cells [3,25,48]. The generation of action potential leads to the release of adenosine 5'-trisphosphate (ATP) through membrane depolarization-dependent channels which is detected by receptors of the taste axons which convey information from the taste cells towards the brain [17,18,25].

Sour and salty taste may be mediated by ion channel-type receptors. Sour taste is clearly initiated by protons acting at the sour taste receptor [32,73,74]. However, interestingly, sour taste intensity is not necessarily proportional to pH [74,75], because, for example, acetic acid is more intensely sour than HCl at the same pH [74,75]. Many candidate receptors have been implicated such as acid-sensing ion channels (ASICs) [76], hyperpolarization-activated cyclic nucleotide-gated potassium channels (HCNs) [73], potassium channels [77,78], 5-nitro-2-(3-phenylpropylamino)-benzoic acid (NPPB)-sensitive Cl^−^ channels [79], and polycystic kidney disease 1L3 and 2L1 heterodimer (PKD1L3/PKD2L1) ion channels [30,47,52]. candidates PKDs (PKD1L3/PKD2L1) and ASICs may be the most promising [1,80], but their role in the sour sensation must be elucidated in future studies [48].

In the case of salt taste, the epithelial sodium ion channel (ENaC) is believed to be the primary receptor [1,48,81] because amiloride, an epithelial sodium channel blocker, reduces drastically taste cell, neural, and behavioral responses to NaCl [48,82,83,84,85,86]. The epithelial sodium ion channel (ENaC) is a Na^+^-specific salt taste receptor [74]. In addition, data indicate the presence of at least one more salt taste receptor that responds to a variety of cations, including Na^+^, K^+^, NH4^+^ and Ca^2+^, and is amiloride insensitive [74,87]. This receptor is distinct from the amiloride-sensitive Na^+^-specific salt taste receptor (ENaC) and are likely to be expressed by different taste cell types [74,87,88]. An amiloride-insensitive and *not* Na^+^-specific components of salt taste responses was suggested to be mediated by a taste variant (TRPV1t) of the transient receptor potential (TRP)-type nonselective cation channel-coupled vanilloid receptor-1 (TRPV1) [48,74,81,89]; however, additional mechanisms may also contribute to the amiloride-insensitive salt taste response [48,81,90].

In the case of sour and salty taste sensitive cells, in which channel type receptors are activated by the taste compounds, the signal transduction occurs by means of the ion channel activation induced depolarizations of these cells, which elicit action potentials that depolarize the taste cell leading to the generation of action potentials by means of the voltage-gated sodium channels (VGSC) of cells [48]. The generation of action potential leads to the release of not yet characterized signaling molecules (may be adenosine 5'-trisphosphate/ATP/or serotonin/5-HT/ [17,18,48,52]) which signal is detected by the taste axons conveying information from the taste cells towards the brain [17,18,48,52].

There are also various receptors of the complementary taste modalities like “fat taste” or “fatty acid taste” [7] “CO_2_ taste” [8] and taste related-sensation “astringency” [9,10]. However, the mechanism of how premised complementary sensing pathways occur has not yet been fully established. The available related data will be introduced together with the action of saliva on these sensing mechanisms in the next chapter. Saliva-related data about fat/fatty acid taste [7] and astringency [10] will be discussed in separate subheadings, whereas the very few available saliva-related data about CO_2_-taste [8] will be mentioned in the subheading dedicated to the salivary carbonic anhydrase (see below).

## 4. Saliva and Taste Perception

### 4.1. General Considerations about Saliva

Saliva is a body fluid, primarily produced by three pairs of major salivary glands (parotid, submandibular and sublingual) and by many minor salivary glands [91,92,93]. Primary saliva is secreted in secretory endpieces (acini) of salivary glands. Primary saliva is modified by serum exudates via tight junctions between several glandular cells (ultrafiltration) and via transcellular diffusion through these cells [91,92,93]. Primary saliva is also modified in the intercalated, striated and excretory (collecting) ducts leading from the acini to the mouth [91,92,93]. The whole secretory processes of the salivary glands (either flow rate, ion secretion or protein secretion) are primarily regulated by their parasympathetic and sympathetic autonomic innervation [94,95].

Entering the mouth, ductal saliva of the salivary glands are blended, and supplemented with various constituents originating from intact or destroyed mucosal cells, immune cells, and oral microorganism [91,92,93]. Importantly, blood constituents also enter the oral cavity via gingival crevicular fluid, through the mucosa as mucosal transudate, and via intraoral bleeding [91,92,93]. Thus a complex mixture of a high variety of molecules results in the oral cavity, frequently called “mixed saliva” and/or “whole saliva” in the scientific literature [91,92,93].

Whole saliva is a major determinant of the environment on all the oral surfaces. On tooth surfaces, saliva plays an important role in acquired pellicle formation, which is a thin layer of several salivary proteins with calciumhydroxide-binding properties [91,92,93]. The acquired pellicle plays a major role in crystal growth homeostasis of the teeth, and in physico-chemical defense of tooth surfaces [91,92,93]. The acquired pellicle also plays a major role in bacterial adhesion (and colonization) on tooth surfaces which may disadvantageously lead to caries formation and periodontal inflammation (especially in the absence of proper oral hygiene) [91,92,93]. Besides defense of tooth surfaces, saliva plays an important role in physico-chemical as well as immune defense of the oral mucosal surfaces (via both direct antimicrobial action and agglutination or surface exclusion of microbes) [91,92,93]. Saliva also plays an important role in the maintenance of oral mucosal structures as well as in the healing of several mucosal lesions, wounds, and ulcers [91,92,93].

Saliva also takes part in the maintenance of the structures of taste buds and taste-sensing cells. In this respect, the effect of salivary epidermal growth factor EGF [96], in addition to the effect of various salivary defense proteins [93], including the salivary molecular chaperone HSP70HSPA family [97,98] should be considered first.

As mentioned above, saliva is primarily produced by three major salivary glands—the parotid, the submandibular and the sublingual glands—and by numerous minor salivary glands [91,92]. In relation with the minor salivary glands, it should be mentioned that the von Ebner’s glands are of particular interest. These glands are contained within the tongue and drain directly into the cleft of the taste buds containing circumvallate and foliate papillae [99]. Their ducts open exclusively into the trough at the base of the papillae, where taste buds open with their taste pores into the trough [100]. Consequently, the taste pores of taste buds are in direct contact with the secretions of von Ebner’s glands in premised (circumvallate and foliate) papillae [100].

The secretory fluid of these glands besides others contains certain hydrophobic molecule transporter (lipophilic−ligand carrier) proteins expected as “tastant-binding proteins” [99,100] and proteins of various other binding abilities [100,101] as, for example, Ebnerin [99]. The hydrophobic molecule transporter “*tastant-binding proteins*” were hypothesized to function as necessary cofactors in taste perceptions by concentrating and delivering hydrophobic sapid molecules (hydrophobic tastants) to the receptors of taste sensing cells [100]. Ebnerin was hypothesized to bind proteins of the surface of taste bud cells and/or to bind various tastants and/or to bind soluble proteins [99] may be including various paracrine regulator proteins present in the fluid within taste buds.

Similar “tastant-binding proteins” and other proteins with various protein-binding abilities are conceivably also present in the whole saliva. It is also likely that whole saliva fulfills all those functions in relation with other taste buds, which are mentioned above in conjunction with the saliva of von Ebner’s gland in the case of taste buds of circumvallate and foliate papillae.

### 4.2. Flow Rate, Buffer Capacity, Ionic Composition

It is likely that the flow rate of saliva can modify the concentration of tastants and various soluble taste perception-related mediators because of a dilution effect [102,103]. Furthermore, the buffering capacity of saliva may also play a significant role in the sensation of sour taste [104], which is strongly (even if not necessarily proportionally [74,75]) coupled with the pH value [102]. However, it should be emphasized that higher flow rate and/or higher salivary buffer capacity does not lead to compromised taste sensation capacity [102]. In fact, it may be quite the contrary: there are available data in the literature that may indicate superiority of taste sensation of subjects with high salivary flow rate compared to those with low salivary flow [102]. Similarly, some data indicate that the perception of bitter and sweet taste is much less affected by flow rate than perception of sour and salty taste [103].

Besides flow rate and buffer capacity, ionic composition of saliva may also play a significant role in taste sensation. Since salt taste of various ions (primarily of Na^+^, but also of others like K^+^, NH_4_^+^ and Ca^2+^) is detected only when above salivary concentrations, saliva influences salt taste threshold levels [104]. Salivary water and electrolytes also influence the ionic environment for taste cells, which is probably critical in taste-related signal transduction [104,105,106]. For example, the potential differences between the cationic/anionic constitutes of the saliva and the fluid present in the taste buds around the taste cells may generate a liquid junction potential which leads to the generation of slow intracellular potentials of taste cells and consequent alteration of taste [107,108]. The most important ions in this relation may be salivary Na^+^, K^+^, Cl^−^, HCO_3_^−^ and the charged salivary proteins [107,108] because the concentrations of these ions (as well as the proteins) are highly increased during nutrition [91] due to the action of masticatory-salivary and gustatory-salivary reflexes [104,109,110].

Salivary zinc ion content was also linked to taste function [111,112,113] because therapeutic administration of zinc could improve certain gustatory dysfunctions due to improvement of the function of the zinc-containing salivary carbonic anhydrase (carbonic anhydrase VI (CA-VI), see also below) [112,113]. This is partially because the enzymatic function of salivary CA-VI depends on the presence of zinc at its active site, but also because zinc treatment can increase salivary CA-VI (also termed “gustin”) concentrations in individuals with certain CA-VI-dependent hypogeusias [114].

### 4.3. Salivary Metabolic Polypeptides

Certain metabolic polypeptides, including leptin [115], ghrelin [116], insulin [117], neuropeptide Y (NPY) [118] and peptide YY (PYY) [119], have been shown to be present in saliva and the cognate receptors for these peptide hormones are expressed in taste cells [1,120,121,122,123]. Anatomical proximity of premised peptides and their receptors suggested their putative roles in taste functions [1,119,121,123]. It is therefore no wonder that these polypeptides have been implicated in modulation of different tastes in general [15,119,123] or more concretely in relation with sweet [120], salty [1,121,122], sour [1,121] and the complementary “fat” [124] taste.

There is increasing evidence that taste perception can be modulated by premised salivary polypepides interacting with their respective receptors expressed in the taste cells; in this way, they influence food intake through fine tuning of taste perception [15,119,123]. However, only the apical parts of the taste cells are exposed to the saliva, because the intercellular barriers of the taste pore of taste buds include tight junctions [15,125] which serve as hard-to-penetrate semipermeable barriers that make taste cells accessible for such polypeptides only at the apical end [15]. Thus, in cases without available concrete evidence in this relation, it should be at least hypothesized that the receptor of a certain salivary metabolic polypeptide is located apically on the targeted taste cells. Alternatively, certain transport mechanisms of these peptides within/through the taste bud cells may be postulated, similarly to the action of certain metabolic polypeptides (*i.e.*, NPY and PYY) on basal oral epithelial cells [123].

#### 4.3.1. Salivary Leptin

Leptin is a 16 kDa hormone first reported as being produced by adipocytes, but it is also expressed by other cell types, including several cells of mammary gland and placenta and also chief cells of stomach [115,126]. Leptin was also detected in major human salivary glands, in the epithelial cells of intralobular ducts [126]. The expression of functional leptin receptor in leptin-producing cells suggests that leptin may exert an autocrine regulatory control of its own synthesis [126].

Leptin acts mainly on the hypothalamus, where it exerts a strong neuroendocrine effect on food intake and energy metabolism, but may also act on several other peripheral organs [115,126] because specific receptors for leptin have been found in various tissues like the thyroid gland, adrenal glands, lung, placenta, kidney, liver, endothelial cells, gastric mucosa and intestinal epithelium [115]. Importantly, leptin receptors were also detected in taste cells [120]; and in mice, a subset of taste receptor cells in tongue epithelium was affected by systemic leptin administration leading to suppressed taste nerve response to sweet stimuli [120], very likely due to a peripheral endocrine effect on functional leptin receptors expressed by taste cells [120].

Besides its endocrine action, leptin may also act in an exocrine manner [126]. Leptin is likely to be produced and surely stored and also secreted into the gastric juice by gastric chief cells [127,128]. Since leptin is not proteolytically degraded [128] and seems to be rather stable under acidic conditions [115], it may have exocrine effects in the stomach and intestine by means of leptin receptors present in the gastric and intestinal epithelium [115,126].

Leptin was also detected in saliva [115,126], and data suggests that it is produced, stored and secreted as an exocrine secretory product [115,126] of the epithelial cells of intralobular ducts [126]. Besides this, a leptin transport from the blood through the salivary glands [115] and/or oral mucosa (*i.e.*, mucosal transudate) may also be possible. Salivary leptin seems to be also rather stable in the oral cavity [115], thereby indicating that leptin may act in an exocrine manner also in the oral cavity. A possible target of salivary leptin may be sweet taste-sensitive cells. Although the function of these cells was shown to be regulated via endocrine action of leptin [120], it may not be excluded that salivary leptin can also act on these cells by means of contamination of the fluid around the taste pores of taste buds.

#### 4.3.2. Salivary Ghrelin

Ghrelin is a 28-amino-acid peptide hormone, and similar to many other peptide hormones, it is processed from a larger precursor (94-amino-acid) by prohormone convertase PC1/3 [1,129]. Ghrelin is a ligand for the growth hormone secretagogue receptor (GHSR); to activate GHSR, ghrelin must be acylated with an eight-carbon fatty acid at serine 3 by ghrelin O-acyltransferase (GOAT) [1,116,130,131,132]; accordingly, the main functions of ghrelin are based on its growth-hormone-releasing activity [116]. GHSR, the main binding site for ghrelin, is produced throughout the brain, as well as in various peripheral tissues in two described isoforms, GHS-R 1a and 1b [116]. Although ghrelin is produced predominantly in the stomach [116], its expression is not limited to the stomach and is found at many other sites such as the small intestine, brain, pituitary, lung, skeletal muscle, islets of Langerhans, adrenal glands, ovary, and testis [133], as well as the kidney and placenta [116]. Ghrelin is also present within the taste buds of the tongue [1], and it has been shown to be produced by human salivary glands and is secreted into saliva [116].

Ghrelin is conventionally considered to be an appetite-regulating hormone. Ghrelin also has many other actions linked to feeding behavior, energy homeostasis, reproduction, sleep regulation, corticotrope secretion and regulation of gastro-entero-pancreatic functions [1,116,134,135]. Ghrelin plays a major role in the gastrointestinal tract, stimulating gastric contractility and acid secretion, and it is responsible for the metabolic response to starvation by modulating insulin secretion, glucose metabolism, and amino-acid uptake [1,116]. Furthermore, it affects cardiovascular activity [116] by acting as a vasodilator. Ghrelin is also likely to influence proliferation processes, though in this case, the data are contradictory [116]. In the case of oral keratinocytes, a proliferative effect of salivary ghrelin is likely [116].

As indicated above, ghrelin is processed from a larger precursor (94-amino-acid) by prohormone convertase PC1/3 [1,129], and both premised precursor and PC1/3 were found to be present within taste cells [121]. Interestingly, GSHR (the cognate receptor of ghrelin) is also expressed in type I, II, III taste cells and, in certain cases, ghrelin and GHSR may colocalize in the same cells [1], thus suggesting that ghrelin may also work in an autocrine manner in these particular cells [1] and may exert an autocrine regulatory control of its own synthesis. In the case of other cells, the effect of ghrelin may be a paracrine effect [1]. GHSR null mice exhibited significantly reduced taste responsivity to sour (citric acid) and salty (sodium chloride) tastants [1], suggesting that ghrelin plays a local modulatory role in salty and sour taste responsivity [1].

As indicated above, to activate GHSR (the cognate receptor), ghrelin must be acylated with an eight-carbon fatty acid at serine 3 by ghrelin *O*-acyltransferase (GOAT) [1,116,130,131,132]; importantly, GOAT is also expressed in all types (*i.e.*, types I, II and III) of taste cells [1]. However, GOAT and ghrelin are not co-localized in all taste cells: a subset of ghrelin immunopositive cells was found to contain no discernable GOAT expression [1]. It was found that 4% of total taste cells were GOAT immunopositive, 13% of total taste cells were ghrelin immunopositive, and both GOAT and ghrelin were co-expressed in 4% of taste cells only [1]. This observation could explain why the GHSR null mice only showed alterations in taste sensitivity to salty and sour tastants, even though ghrelin and GHSR were expressed in all types (*i.e.*, type I, II and III) of taste cells [1].

Ghrelin and the two receptor isoforms, GHS-R 1a and GHS-R 1b, are also produced by the human salivary glands, with subsequent excretion of the hormone into saliva [116]. Concentrations of salivary ghrelin were lower than those in serum with a significant correlation between both body fluids [116]. Stimulation of the salivary flow rate with citric acid led to significantly decreased ghrelin concentrations [116], indicating that excretion of ghrelin into the saliva is either due to a transport from the blood vessels into the glandular cells [116], or due to a small capacity secretion from the salivary glands (or both). Production of ghrelin mRNA was found in all major salivary glands [116], and immunohistologic staining indicated ghrelin with granular concentration near the cell membranes of the ducts; which may indicate storage of ghrelin within the glands’ ductal cells before release into the ductal lumen. Besides blood and salivary glands, oral keratinocytes may also be an additional source of salivary ghrelin, because production of ghrelin mRNA was found not only in all major salivary glands [116], but also in oral keratinocytes [116].

Importantly, there are both acylated and des-acylated ghrelin in saliva [116]; latter may be because of limited stability of the acylated form of salivary ghrelin [116]. However, it was assumed that the des-acylated ghrelin may even counteract certain metabolic (but not neuroendocrine) functions of the acylated portion [116,136]. Thus, it seems to be a simplification to classify salivary ghrelin into “active” (*i.e.*, acrylated) and “nonactive” (*i.e.*, des-acrylated) forms [116], in relation with the expectable effect of salivary ghrelin on taste. Ghrelin present in the saliva is likely to contaminate the fluid present around the taste pores of taste buds [1], and as such it may take part in the fine tuning of salty and sour taste perception getting contact with the apical region of taste cells. The ratio between the acylated and des-acylated form of salivary ghrelin may also have certain effects on this fine tuning.

#### 4.3.3. Salivary Insulin

Insulin is the main hormone controlling carbohydrate and lipid metabolism, produced by pancreatic B-cells of the islets of Langerhans [15]. Insulin-like immunoreactivity has been reported in the major salivary glands [137,138], and insulin was also found in human saliva [117,139]. It is likely that salivary insulin arrives into the saliva from the blood via ultrafiltration [117,139,140]; however, a certain amount of local synthesis and secretion of insulin in the salivary glands should not be excluded [15]. The expression of insulin receptors have been shown in taste cells in murine [122], and data indicate that salivary insulin plays a role in the control of salty taste modality through acting on these peripheral insulin receptors [122]. It is not yet clear that if there are apically located insulin receptors of taste cells; consequently, it cannot be ruled out that salivary insulin also takes part in the fine tuning of salty taste by means of apically located insulin receptors.

#### 4.3.4. Salivary Neuropeptide Y (NPY3-36) and Peptide YY (PYY3-36)

Neuropeptide Y (NPY) and peptide YY (PYY) belong to a family of peptides sharing similar hairpin-like PP-fold structural homology and evolutionary history [123,141]. NPY is widely expressed in the central as well as in the peripheral nervous system while PYY is released mostly by l-endocrine cells in the distal gut epithelia [119,123,141]. These peptides mediate various complementary and often opposing metabolic functions like appetite and satiation; energy intake and expenditure; cell proliferation, migration, and differentiation; neuromodulation, angiogenesis, osteogenesis; and many other biological processes [119,123,141]. This diversity of functions is mediated through PP-fold peptide binding receptors (YRs) referred to as Y1R, Y2R, Y4R, Y5R, and y6R [123,141]. Importantly, YRs are also present in various oral mucosal tissues including taste cells [119,123].

Significantly, both neuropeptide Y (NPY) [118] and peptide YY (PYY) [119] are present in human saliva, and both are likely to originate from two independent sources such as circulating plasma (*i.e.*, transudate from blood) and taste bud cells [119,142]. In the saliva, a serine exopeptidase (salivary dipeptidyl-peptidase-IV/DPP-IV/secreted from salivary glands [143,144]) truncates NPY and PYY at their N termini producing truncated peptides NPY_3–36_ and PYY_3–36_ and thereby changing their binding specificity to YRs [119,123]. However, premised truncated forms are not specific to saliva [119,123] because the dipeptidyl-peptidase-IV (DPP-IV) is also present in the plasma [144,145] and may also be produced in other tissues [144].

It is significant that all tested YR subtypes (Y1R, Y2R, Y4R, Y5R) are prominently expressed in mice taste cells and showed preferential apical distribution within most of the cells [119,123]. This distribution would make YRs easily accessible not only to paracrine NPY and PYY (originating from taste bud cells) but also to salivary NPY_3–36_ and PYY_3–36_ peptides, thereby suggesting the possible role of salivary NPY_3–36_ and PPY_3–36_ in modulating taste perception. YRs were colocalized primarily with those neuronal markers which are expressed in type II cells [124], and to a lesser extent also with those expressed in type III cells [123,124]. It is thus conceivable that salivary NPY_3–36_ and PYY_3–36_ peptides may play a role in the modulation of one or another type III cell-dependent taste modalities (*i.e.*, sweet and/or umami and/or bitter), and perhaps also in the modulation of sour taste.

Available data point to salivary PYY_3–36_ likely *not* being involved in the modulation of bitter taste [15,124], whereas NPY_3–36_ is likely to be involved in the modulation of sweet taste [15]. Interestingly, salivary PYY_3–36_ is likely to play a role in the modulation of a complementary taste modality referred to as fat/fatty acid taste [15,124]. In recent animal studies, an increase in *salivary* PYY_3–36_ resulted in a significant reduction in food intake providing further evidence for a complementary oral pathway of action of PYY_3–36_ [15,119,124] (and expectably also of NPY_3–36_).

### 4.4. Glucagon-Like Peptide-1 (GLP-1)

Glucagon-like peptide-1 (GLP-1) is typically considered as a hormone produced by the endocrine cells of the gut [1,121]. Its primary peripheral function is to regulate insulin secretion and gastric emptying [1,121]. GLP-1 is also involved in a wide range of other physiological functions, including the food intake and control of body weight, neuroprotection and neuronal regeneration in the CNS, learning and memory, cardiac function, and bone resorption [43].

Importantly, GLP-1 is produced in sweet and umami taste-sensing cells [1,43,121], and the enzyme prohormone convertase 1/3 (PC1/3), which cleaves pro-glucagon into GLP-1, is also present within these subsets of taste cells [1,43,121]. Since the GLP-1 receptor (GLP-1R) is expressed on taste nerve fibers found in close proximity to GLP-1-containing (sweet and umami sensing) taste cells. [1,43,121], it is very likely that GLP-1 is not only produced in but also released from these taste cells and acts on taste nerve fibers in a paracrine manner. Accordingly, disruption of GLP-1 signaling causes a significantly decreased sensitivity to sweet tastants, and increased sensitivity to umami tastants in mice [1,43,121]. There was also a modest increase in citric acid taste sensitivity following disruption of GLP-1 signaling [1,43,121]; thus it is possible that GLP-1 also modulates sour taste [1,43,121]. These findings indicate that GLP-1 plays a role in the fine tuning of sweet, umami and maybe also of sour taste perception in a paracrine manner.

In contrast to the other metabolic polypeptides discussed above, GLP-1 was not yet found in the human whole saliva [140]. It is regardless a possibility that there is a certain amount of GLP-1 in the excretion of particular major or minor salivary gland(s) [91,92]—including the von Ebner’s glands—that may be present locally as mucosal transudate [91,92]. In this relation, saliva secreted by the von Ebner’s salivary glands may be of particular importance, as these glands drain directly into the cleft of the circumvallate and foliate papillae [99] (which contain high number of taste buds). The secretory fluid of these glands surely gets in contact with the apical region of taste cells because the ducts of these glands open into the trough at the base of the papillae, where taste buds open with their taste pores into the trough [100].

The hypothesis that a certain amount of GLP-1 may be present in the saliva, or at least around certain taste buds, is supported by the finding that local oral (oral spray) administration of GLP-1 (as well as its homolog, Exendin-4) induced reduction of food intake in murine very likely via direct action on GLP-1 receptors of taste cells [15,119]. Thus, it may not be excluded that there is also an oral pathway of GLP-1-dependent fine tuning of taste sensation.

### 4.5. Salivary Carbonic Anhydrase (CA-VI, Gustin)

The carbonic anhydrases (CAs) are an expanding family of zinc-containing enzymes, which participate in the maintenance of pH homeostasis in the human body, catalyzing the reversible reaction: CO_2_ + H_2_O ↔ HCO + H [146]. Carbonic anhydrase VI (CA-VI) is the only known secreted isoenzyme of this family [146,147], which has been detected in the saliva [148,149,150]. CA-VI is secreted by the serous acinar cells of mammalian parotid and submandibular glands [151,152], as well as by the von Ebner’s gland [153], and it has been proposed that this isoenzyme may participate in protecting the teeth from caries [154] and in neutralizing excess acid in the mucous layer covering the esophageal and gastric epithelium [146,147] (*i.e.*, a high amount of salivary CA-VI is supplied to the gastrointestinal tract together with swallowed saliva). CA-VI was also linked to taste function because a previous study reported that hypogeusic subjects had salivary CA-VI as low as 20% of that of normal subjects [114]. Although CA-VI can alter pH and as such may influence sour taste, CA-VI gene-related studies confirm that salivary CA-VI is primarily involved in bitter taste perception [7,147,155].

Importantly, CA-VI was identified as gustin [156], a previously recognized zinc-binding salivary protein linked to the regulation of taste function [112,113,114]. Gustin was first implicated in growth and maintenance of taste buds [112,113,114]; however, direct evidence that gustin can act as a trophic factor that promotes the growth, development and/or maintenance of taste buds has been lacking. Recently, data indicate that gustin play a major role in the development and maintenance of the fungiform taste papillae [157]. Interestingly, gustin was also linked with the recognition of a complementary taste modality termed fat/fatty acid taste, through its effect on bitter taste (*i.e.*, 6-*n*-propylthiouracil/PROP/) recognition [7,158,159,160]. Moreover, a sour taste cell membrane-associated form of CA-VI was shown to function as receptor of a complementary taste modality referred to as CO_2_ taste [8]. Higher salivary concentration of salivary carbonic anhydrase (CA-VI) was also associated with a lower bitterness acceptance (expectably increased bitter taste perception) in infants [161], indicating that this protein is likely to be involved in the fine tuning of bitter taste perception already in infants.

### 4.6. Salivary Proline-Rich Proteins (PRPs)

Proline-rich proteins (PRPs) form a major fraction of salivary proteins. The PRPs are highly phosphorylated proteins [162]. The various PRPs are encoded by seven genes. Many of the PRPs are subsequently cleaved by proprotein convertases before secretion, thus leading to a large number (more than 20) of PRPs in the saliva. The molecular weight of acidic and basic PRPs is usually between 10 and 40 kDa, whereas the large glycosylated PRPs have a molecular weight of 60–70 kDa [163]. A major source of salivary proline-rich proteins (PRPs) are the salivary glands [91,93]; the highest concentration of PRPs was found in the parotid saliva [164].

Acidic PRPs contain a longer and highly acidic *N*-terminal region, and a somewhat different repeated sequence compared to basic PRPs [162]. Acidic PRPs exert calciumhydroxide-binding properties and therefore participate in the formation of an acquired pellicle (a thin protein layer) on the surfaces of teeth [91,93,165]. Basic PRPs are also present in the human-acquired enamel pellicle [165]. Acidic PRPs bind bacteria and basic PRPs bind fungi (e.g., Candida albicans) and viruses, whereas glycosylated PRPs bind bacteria and viruses, thereby indicating the role of PRPs in the clearance towards the stomach and/or surface exclusion of these microorganisms [91,93,166,167]. PRPs are also potent precipitators of various polyphenols including tannins [162].

Salivary PRPs were expected to play a role in the perception of astringency (see below) based on their premised high polyphenol-binding affinity [10,168,169] and on the high lubricating properties of glycosylated PRPs [10,168]. Salivary PRPs were shown to be inducible (*i.e.*, were upregulated in saliva) in rats exposed to a tannic acid diet [169], and their induction was significantly correlated with higher acceptance of licking tannin-containing solutions [169]. These data also suggest a role for salivary PRPs in the perception of astringency.

Importantly, salivary PRPs were also recently linked with bitter taste recognition [155,170]. Studies in relation with PROP (6-*n*-propylthiouracil)-induced bitter taste sensation indicate that the salivary level of certain members (namely Ps-1 and II-2) of the basic proline-rich protein family were higher in the unstimulated saliva of PROP super-tasters and PROP medium tasters compared to PROP non-tasters [155,170]. Data also indicated that PROP stimulation elicited rapid increase in the levels of these same proteins only in the saliva of PROP super-tasters [155]. Furthermore, supplementation of Ps-1 protein in those non-taster individuals lacking Ps-1 in their saliva enhanced their PROP bitter taste responsiveness [170]. Premised data strongly indicate the role of PRPs (or at least of certain PRPs) in bitter taste perception.

### 4.7. Salivary Cystatins

Cystatins are cysteine protease inhibitors that block the action of endogenous, bacterial and parasitic protozoan proteases [171,172]. Cystatins also exert direct immunomodulatory properties and likely certain antiviral effects, too [171]. The human cystatin gene family contains 14 genes (including two pseudogens) from which seven cystatins are present in saliva [172], namely cystatin-A, cystatin-B, cystatin-C, cystatin-D, cystatin-S, cystatin-SA and cystatin-SN [172]. The highest concentration of cystatins was found in the submandibular saliva [164], but (in much less concentration) they are also present in the parotid saliva [171]). Cystatins are also present in the gingival crevicular fluid [173]. Cystatin-SN and cystatin-S are also present in the human-acquired enamel pellicle [165] and also bind bacteria as well as bacterial lipopolysaccharides (LPS) [174].

Although data are still contradictory as to whether increased [175] or decreased [176] concentration of salivary cystatin-SN is coupled with improved bitter taste sensation, there is increasing evidence that cystatin-SN plays an important role in bitter taste sensation [10,168,175,176]. It was also hypothesized that an important aspect of the effect of cystatin-SN may be based on its protease-inhibitory function [176]. Higher bitter taste acceptance (expectably decreased bitter taste perception) of infants was also associated with higher salivary concentration of cystatin-S in a study, indicating that not only cystatin-SN but other type of cystatins are also likely involved in the fine tuning of bitter taste perception [161]. Salivary cystatins were also expected to play a role in the perception of astringency (see below) based on their high polyphenol-binding affinity [10,168]. Salivary cystatin-S was shown to be inducible (*i.e.*, were upregulated in saliva) in rats exposed to atannic acid diet [169]; its induction was significantly correlated with higher acceptance of licking tannin-containing solutions [169]. These data also suggest a role for salivary cystatin-S in the perception of astringency.

### 4.8. Saliva and Fat/Fatty Acid Taste

Historically, the perception of dietary fat was thought to be based on olfactory and textural (e.g., oiliness and viscosity) properties [14]; however, when these properties are masked, both animals and humans are still able to distinguish between fatty acids and control solutions as well as between various fatty acids [14,177,178]. Importantly, humans are likely able to detect both fat in general [14] and a range of free fatty acids that vary in saturation and chain length even if visual, olfactory, and textural differences in the samples are controlled [178]. All these findings are especially important if we consider that related studies in humans strongly indicate that fat-sensing mechanisms may contribute to overeating and obesity [11,12,13,14,15].

The mechanism how fat/fatty acid-sensing occurs has not yet been established. There are various candidate fatty acid receptors expressed in taste cells, like fatty acid translocase CD36 and G protein-coupled receptors GPR126 or GPR40 [6,124]. One candidate oral fat sensor that has received considerable attention is the fatty acid translocase CD36, which is homologous to fatty acid transporter in animals [14]. CD36 is an 88 kDa membrane-bound protein that is expressed in multiple cell types and has a broad range of functions in immunity, inflammation, and lipoprotein metabolism [179]. It is also involved with the transport of long-chain fatty acids across cell membranes, a first step in fat metabolism [179]. It is significant that CD36 is also expressed on taste cells in animals [6,180,181] and humans [6,181]. Importantly CD36 is located apically on taste cells [180,181], and is therefore available for both tastants and saliva constitutes.

It is very likely that CD36 is involved in the recognition of fatty acids in the oral cavity [6,182], and available data indicate that CD36 is also required to show preferences for triglycerides [6,14,183]. These data suggest that CD36 is not only involved in recognition of various fatty acids but also in the detection of “fats” in general [14]. This is because in naturally occurring fats the proportion of triglycerides rarely contains the same fatty acid residue in all three ester positions. In contrast, the proportion of triglycerides in naturally occurring fats contains a various mixture of fatty acids [184]. Thus, even if general fatty acid composition, processing, and source can influence the overall flavor of naturally occurring fats [14], their predominant sensory property is simply “fatty” [14].

Available data clearly indicate the importance of saliva in fat/fatty acid taste sensation [185]. Although the concrete mechanism are not clearly understood yet, it is very likely that, salivary PYY_3–36_ (about PYY_3–36_ see also above) plays an important role in the modulation of a fat/fatty acid taste [15,124]. It is hypothesized that the saliva-mediated fine tuning of oral fat/fatty acid sensation occurs due to the action of salivary PYY_3–36_ on Y2R type PP-fold peptide-binding receptors (Y2Rs) localized at the apical parts of taste cells [124]. Salivary carbonic anhydrase (CA-VI, gustin, see also above) was linked with the recognition of a complementary taste modality termed “fat-taste” or “fatty acid taste,” through its effect on bitter taste (*i.e.*, 6-*n*-propylthiouracil/PROP/) recognition [7,158,159,160]. This is because available data indicate that PROP nontasters have a lower ability to distinguish fat content in foods and show higher acceptance of dietary fat than tasters [7,158,159,160].

### 4.9. Saliva and Astringency

Astringency is a *tactile* sensation [9,10] described as the drying and puckering of the oral surface experienced when ingesting polyphenol-rich plant foods and beverages. Although astringency is not a real taste, sensing it strongly interferes with real taste-sensing phenomena, especially bitter, sour and sweet taste sensing [16]. Therefore, it can be considered as a rather important complementary modality of taste sensing. It has been proposed that the sensation of astringency represents a sensory warning cue that would discourage the ingestion of foods that contain too many polyphenolic compounds [10,16,168].

It is generally accepted that key steps of astringency elicitation are phenol/salivary protein interactions [9,186], leading to stimulation of mechanoreceptors by precipitated salivary proteins [187,188] and/or rupture of the lubricating saliva film that lines the oral cavity [189,190]. In this respect, a two-phase saliva/polyphenol interaction was also hypothesized [191] in which the first phase of interaction involves the precipitation of proteins with the highest phenol-binding affinity followed by the interaction of polyphenols with the surface-adsorbed glycoprotein layer as a second phase leading to consequent oral cavity delubrication and astringency elicitation [191].

Thus, it is generally well accepted that the gradual lowering of (soluble and/or surface-attached) polyphenol-precipitating proteins under polyphenol exposure implies an increasing involvement of the deeper mucosal surface-attached protein layer, leading to the rupture of the lubricating film and the consequent appearance of an astringency sensation [10,191]. Accordingly, it can be assumed that the sensation of astringency strongly depends on the presence of salivary proteins of the highest phenol-binding affinity like proline-rich proteins (PRPs), salivary alpha-amylases, cystatins, and histatins [10,162,168,169] as well as on the presence of salivary proteins of high lubricating properties like alpha-amylase, glycosylated PRPs [10,168] and salivary mucins. On the other hand, it is also likely that not exclusively absolute values of premised salivary proteins but also differences between basal and stimulated oral conditions are highly important for astringency phenomena [10]. For example, a decrease of these proteins due to prolonged stimulation could induce a lowering of the usual level of mouth lubrication (and appearance of astringency) also in subjects with a well-lubricated oral environment under rest (*i.e.*, unstimulated) conditions [10,168].

In good accordance with this, data indicate that the ability to maintain a nearly constant protein concentration and an unchanged capacity to bind and precipitate polyphenols after masticatory and/or taste stimulation(s), characterize subjects with lower sensitivity to astringency, whereas a strong reduction in the values of both these salivary characteristics characterize more sensitive subjects [10,168]. Furthermore, it is also likely that maintained concentration of certain salivary proteins of lubricating properties, like α-amylase, glycosylated PRPs after masticatory and/or taste stimulation(s), also characterize subjects with lower sensitivity to astringency as not characterized by sensitive subjects [10]. Although salivary mucins surely also play an important role in lubricating oral surfaces (and as such in the astringency sensation), salivary mucins appear to be maintained in both high- and low-sensitivity subjects following stimulation [10].

### 4.10. Other Salivary Effects on Taste

Whole saliva and also salivary albumin and mucin are able to solubilize lipophilic plant polyphenols [192,193] which are poorly soluble in water and are therefore hardly available for taste cells. Saliva as whole, as well as premised salivary proteins (*i.e.*, salivary mucins and salivary albumin), is likely able to increase the availability of lipophilic polyphenol tastants for taste cells, and in this way can significantly improve taste sensation.

Increased concentration of certain salivary proteins such as salivary alpha-amylase, salivary albumin and salivary immunoglobulin-A (sIgA) also seems to be coupled with improved taste perception namely in relation with the bitter taste sensation [176].

Lower bitter taste acceptance (expectably increased bitter taste perception) of infants was also associated with higher salivary concentration of the secretory component (*i.e.*, for sIgA) and zinc-α-2-glycoprotein [161], whereas higher bitter taste acceptance (expectably decreased bitter taste perception) was associated with higher concentration of salivary lactoperoxidase and prolactin-inducible protein in a study [161], thereby indicating that premised salivary proteins are likely to be involved in the fine tuning of bitter taste perception.

The influence of the 70 kDa salivary molecular chaperone HSP70/HSPAs [194,195] on receptor binding of major umami taste-inducer glutamate to umami taste receptors of taste cells in the mouth was also hypothesized [196]. Considering the “multi target” character of the chaperoning/protein-repair function of salivary HSP70/HSPAs, it may also not be excluded that the salivary HSP70/HSPAs plays important role in the maintenance of taste cells and their taste receptors [97,98].

## 5. Conclusions

The gustatory system plays a critical role in determining food preferences and food intake [1] and thus also in nutritive, energy and electrolyte balance. Fine tuning of the gustatory system is also crucial in this respect. The exact mechanisms that fine tune taste sensitivity are currently poorly defined, but it is clear that various effects of saliva on taste recognition are also involved.

This is especially true of those metabolic polypeptides present in the saliva which have traditionally been considered gut and appetite hormones (*i.e.*, leptin [115]. Ghrelin [116], insulin [117], neuropeptide Y (NPY) [118] and peptide YY (PYY) [119]) were considered as playing a pivotal role in fine tuning [15,119,123].

Moreover, the data clearly indicate the major role of several other salivary proteins: salivary carbonic anhydrase (CA-VI, gustin) [7,8,114], salivary proline-rich proteins (PRPs) [10,155,169,170], salivary cystatins [10,169,175,176], salivary alpha-amylases [10,169], salivary histatins [10,169], salivary albumin [192,193] and salivary mucins [192,193].

A role of certain other proteins like glucagon-like peptide-1 (GLP-1) [1,121], salivary immunoglobulin-A (sIgA) [161,176], zinc-α-2-glycoprotein [161] salivary lactoperoxidase [161], salivary prolactin-inducible protein [161] and the 70 kDa salivary molecular chaperone HSP70/HSPAs [196] may also be expected. Furthermore, factors like salivary flow rate [102,103], buffer capacity [104] and ionic composition [104,106,108,113] of saliva should also be considered.

Although it was suggested nearly a hundred years ago that salivary composition might be responsible for taste differences among people [197], the exact mechanisms of how saliva influences and fine tunes taste sensation is still a challenging field of research. Gaining a greater understanding of which relating factors are present in the saliva, their putative roles in taste bud signaling and overall taste perception will shed some much needed light on how taste sensitivity is fine-tuned and how taste perception is linked to saliva and salivation. In this paper, the most important available data related to this emerging field of research was reviewed.
